# L-arginine supplementation and thromboxane synthase inhibition increases cerebral blood flow in experimental cerebral malaria

**DOI:** 10.1038/s41598-019-49855-x

**Published:** 2019-09-20

**Authors:** Aline S. Moreira, Vanessa Estato, David C. Malvar, Guilherme S. Sanches, Fabiana Gomes, Eduardo Tibirica, Cláudio Tadeu Daniel-Ribeiro, Leonardo J. M. Carvalho

**Affiliations:** 1 Laboratory of Malaria Research, Instituto Oswaldo Cruz, Fiocruz, Rio de Janeiro, Brazil; 2 Laboratory of Immunopharmacology, Instituto Oswaldo Cruz, Fiocruz, Rio de Janeiro, Brazil; 3 Department of Physiological Sciences, Federal Rural University of Rio de Janeiro, BR 465/Km 07, 23897-000 Seropédica, Rio de Janeiro, Brazil; 4 National Institute of Cardiology, Rio de Janeiro, Brazil

**Keywords:** Infection, Pathogens

## Abstract

Cerebral malaria pathogenesis involves vascular dysfunction with low nitric oxide (NO) bioavailability, vasoconstriction and impaired vasodilation, leading to ischemia, tissue hypoxia and ultimately death. Cerebral blood flow (CBF) involves NO and other pathways, including arachidonic acid (AA)-derived metabolites. Here we show that mice with experimental cerebral malaria (ECM) by *P. berghei* ANKA showed marked decreases in CBF (as assessed by laser speckle contrast imaging - LSCI) and that administration of L-arginine supplementation (50 mg/kg) and/or of the thromboxane synthase inhibitor Ozagrel (100 mg/kg) induced immediate increases in CBF. L-arginine in combination with artesunate (32 mg/kg) induced immediate reversal of brain ischemia in the short-term (1 hour), but the effect subsided after 3 and 6 hours. Neither L-arginine nor Ozagrel reversed blood brain barrier breakdown. Mice with ECM showed brain levels of selected AA-derived metabolites with a vasoconstrictor profile, with increased levels of 8-isoprostanes, 20-HETE and 14,15-DHET, whereas mice infected with a non-ECM-inducing strain of *P. berghei* (NK65) showed a vasodilator profile, with normal levels of 20-HETE and 14,15-DHET and increased levels of PGE2. L-arginine is capable of partially reversing cerebral ischemia and AA metabolites may play a role in the cerebrovascular dysfunction in ECM.

## Introduction

Cerebral malaria, a deadly neurological complication of *Plasmodium falciparum* infection, shows features of a severe vasculopathy, which results in impaired perfusion, ischemia and hypoxia^1–4^. In addition to microvascular congestion, endothelial dysfunction has been shown to occur in severe malaria possibly contributing to ischemia^5^. Patients with severe malaria show low nitric oxide (NO) bioavailability resulting in impaired microvascular function^6,7^. Low levels of the NO synthase (NOS) substrate L-arginine and of the NOS cofactor tetrahydrobiopterin are also observed^8–10^, as well as disruption of NOS substrate/inhibitor homeostasis, all these factors limiting NO production^11,12^. These findings prompted the proposal of L-arginine supplementation as a tool to improve vascular function in severe malaria and a number of safety and efficacy studies have been conducted in recent years^13–15^.

Vascular congestion and endothelial dysfunction are also observed in experimental cerebral malaria (ECM) by *Plasmodium berghei* ANKA infection in susceptible mice. Low NO bioavailability with hypoargininemia have been shown to be associated with pathogenesis^16^, and administration of exogenous NO donors prevented the neurological syndrome, ameliorating vascular function and decreasing vascular inflammation and congestion^16,17^. ECM is associated with marked cerebral ischemia caused in part by widespread cerebrovascular constriction, which was ameliorated and even reversed with vasodilator drugs such as nimodipine and glyceryl trinitrate, when given as adjuvant therapy combined with artemisinin derivatives^18–21^. Moreover, pial vessels of mice with ECM present impaired vasodilator responses to acetylcholine and N-methyl-D-aspartate (NMDA), which induce vasodilation through mechanisms involving endothelial and neuronal NOS (eNOS and nNOS), respectively, indicating that both NO-producing enzyme isoforms are dysfunctional in ECM^22^. Importantly, cerebrovascular dilation was restored with direct L-arginine superfusion in pial vessels^23^, and systemic administration of L-arginine at various doses were shown to partially reverse or prevent the aggravation of cerebral vasoconstriction in ECM, and in association with artesunate increased survival of mice with late stage disease^23^.

NO is a central and ubiquitous modulator of vascular tone, but regulation of cerebral blood flow (CBF) is highly complex and involves a number of vasoactive molecules that control blood supply to the different areas of the brain according to demand, in a close interplay with NO biology^24,25^. Among these pathways, the balance of vasoactive arachidonic acid (AA) metabolites plays important roles in CBF regulation. Under hypoxic and pro-oxidant conditions, AA may undergo non-enzymatic oxidation and generate potent constrictive metabolites such as isoprostanes^26,27^. In addition, AA is the substrate for several enzymes that generate vasoactive metabolites, which may have either vasodilator (e.g., prostaglandin E2 [PGE2], epoxyeicosatrienoic acid [EET]) or vasoconstrictor (e.g., 20-hydroxyeicosatetraenoic acid [20-HETE], thromboxane A2 [TxA2], leukotrienes) activities. Therefore, cerebrovascular constriction in ECM could be at least in part explained by excessive generation of AA metabolites with a vasoconstrictor profile. In human malaria, lower levels of the vasodilator PGE2 correlated with disease severity^28^, and reduced PGE2 was shown to occur through hemozoin-induced inhibition of cyclooxygenase-2 (COX-2) in mononuclear cells via an interleukin-10-dependent mechanism^29^. In ECM, sick mice had increased phospholipase A2 mRNA expression in the spleen, and treatment of mice with the prostaglandin synthesis inhibitor aspirin was detrimental^30^. Also, increased COX-2 expression in the brain was observed in mice with ECM, but not in malaria-infected mice with no neurologic involvement^31^.

In the present study we investigated two different and interrelated approaches to reverse cerebral ischemia in mice with late-stage ECM, asking: (a) whether the described effect of L-arginine in dilating cerebral vessels or preventing the aggravation of vasoconstriction indeed results in substantial increases in cerebral blood flow (CBF); (b) whether the pharmacological inhibition of the synthesis of the potent AA-derived vasoconstrictor thromboxane A2, alone or in combination with L-arginine supplementation, results in increased CBF in ECM, as a first effort to address the role of AA metabolites in ECM cerebrovascular dysfunction. We also determined the levels of relevant AA metabolites (8-isoprostanes, TxA2, 20-HETE, PGE2, and 14,15-dihydroxy-5Z,8Z,11Z-eicosatrienoic acid [14,15-DHET]) in the brain of mice with ECM or infected with a *P. berghei* strain (NK65) that does not cause ECM.

## Results

### Parasitemia and temperature

ECM mice that were used in the experiments were hypothermic, with rectal temperatures between 31 °C and 36 °C. At the time of ECM, mice showed parasitemia ranging from 4.0% to 29.5%. In each experiment, rectal temperature and parasitemia did not differ between groups.

### Mice with ECM show decreased cerebral blood flow (CBF)

**Figure 1:**
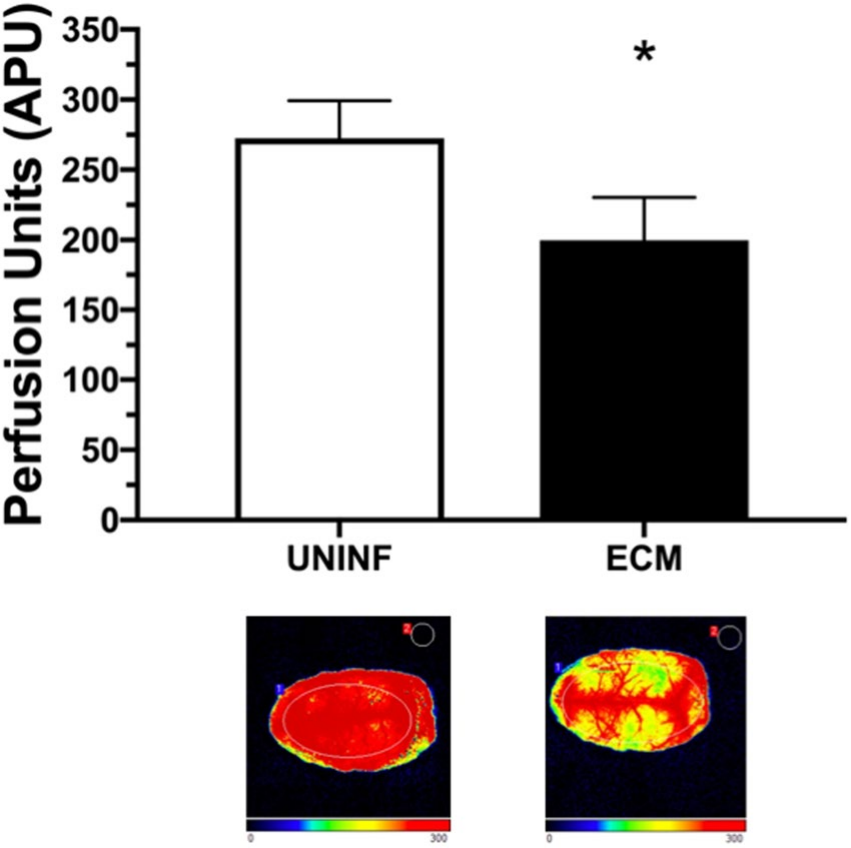
Cerebral blood flow (CBF) of mice with experimental cerebral malaria (ECM) and uninfected controls. CBF (measured as arbitrary perfusion units, APU) of mice with ECM (n = 16) and uninfected control mice (UNINF, n = 11). Mice were anesthetized with urethane, kept at 36 °C, had the skull exposed and were subjected to laser speckle contrast imaging (LSCI) measurements of CBF. Mice with ECM showed significant decreases in CBF (p < 0.0001). Data are presented as mean ± standard deviation. Images at the bottom are representative of CBF for each group as acquired by LSCI.

In line with our previous studies using intravital microscopy^19,32^, mice with ECM presented a marked decrease in CBF (mean: 199.9 ± 30 APU) compared to uninfected controls (mean: 272.5 ± 27 APU), as verified by LSCI (Fig. 1).

### Treatment with L-arginine increases CBF in mice with ECM

**Figure 2:**
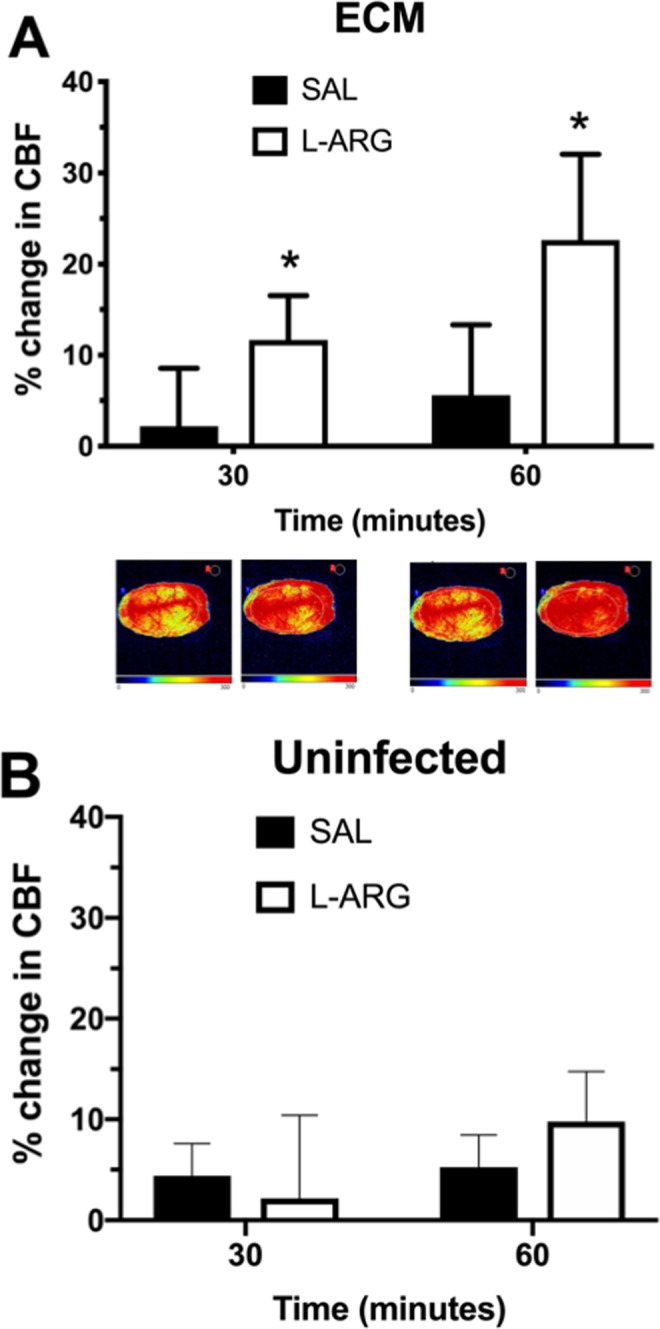
Cerebral blood flow (CBF) of mice with experimental cerebral malaria (ECM) or uninfected mice treated with L-arginine or saline. Percent change in CBF of mice with ECM (**A**) or uninfected mice (**B**) treated with L-arginine (L-ARG) 50 mg/kg or vehicle (saline – SAL), 200 μL, subcutaneously. Mice were anesthetized with urethane, kept at 36 °C, had the skull exposed and were subjected to laser speckle contrast imaging (LSCI) measurements of CBF before and continuously after dosing, for 60 minutes. Mice with ECM that received L-arginine (n = 5) showed higher increases in CBF than mice that received saline (n = 6), at 30 and 60 minutes (p = 0.0071). Data are presented as median ± interquartile range. Images at the bottom are representative of CBF for each group as acquired by LSCI. Uninfected mice that received either L-arginine (n = 4) or saline (n = 4) showed increased CBF at 60 minutes, however the difference between groups (L-arginine and saline) was not significant.

We have previously shown that systemic administration of L-arginine at different doses prevents aggravation and may even partially reverse cerebrovascular constriction in ECM^23^. We asked whether this beneficial effect on pial vessels actually results in increased CBF as measured by LSCI. Indeed, administration of L-arginine 50 mg/kg resulted in a median 26.2% (IQR: 9–31%) increase in CBF after 60 minutes, compared to a median 5.0% (IQR: -7–16%) increase in mice that received saline (Fig. 2A). The effect of treatment in mice with ECM was more pronounced than the effect observed after administration of L-arginine to uninfected mice, which led to a median 8.7% (IQR: 5.3–16.4%) increase in CBF after 60 minutes (Fig. 2B).

### Treatment with the thromboxane synthase inhibitor Ozagrel increases CBF in mice with ECM

**Figure 3:**
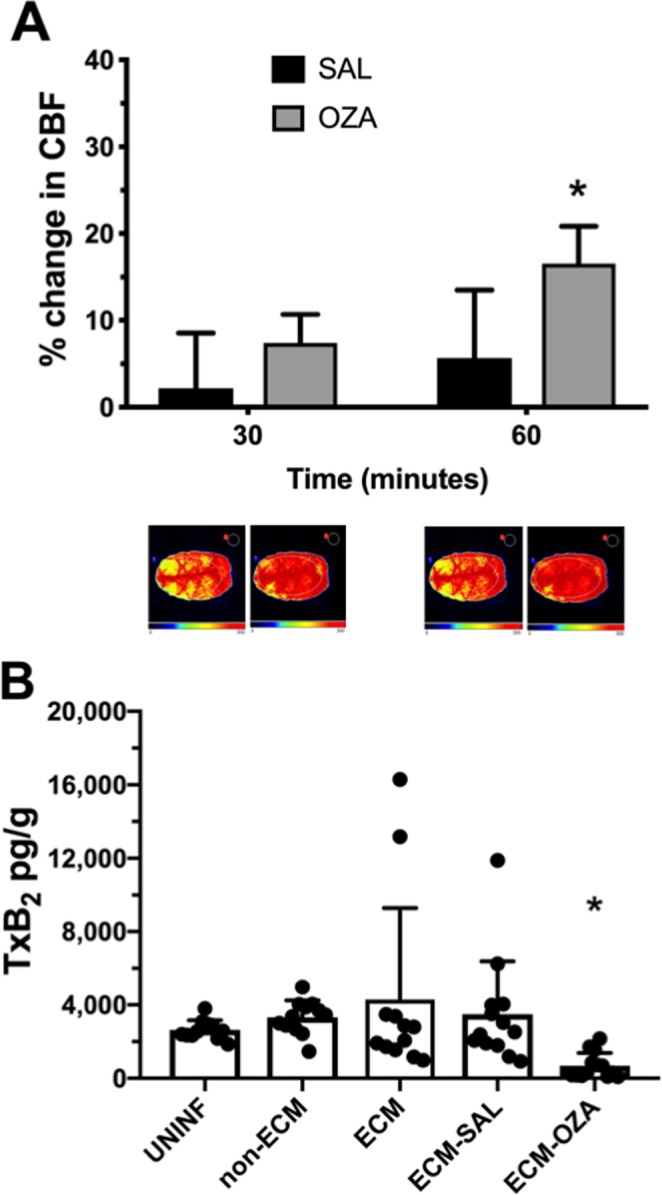
Cerebral blood flow (CBF) and thromboxane A2 levels (measured by detection of its derivative TxB2) in mice with experimental cerebral malaria (ECM) treated with Ozagrel or saline. (**A**) Percent change in CBF in mice with ECM treated with the thromboxane synthase inhibitor Ozagrel (OZA) 100 mg/kg or vehicle (saline – SAL), 200 μL, subcutaneously. Mice were anesthetized with urethane, kept at 36 °C, had the skull exposed and were subjected to laser speckle contrast imaging (LSCI) measurements of CBF before and continuously after dosing, for 60 minutes. Mice with ECM that received Ozagrel (n = 6) showed higher increases in CBF than mice that received saline (n = 6), at 60 minutes (p = 0.032). Data are presented as median ± interquartile range. Images at the bottom are representative of CBF for each group as acquired by LSCI. (**B**) Thromboxane B2 (TxB2, a stable metabolite of TxA2) levels in the brains of uninfected mice (UNINF), mice infected with *P. berghei* NK65 (non-ECM), mice with ECM untreated (ECM), and after one hour of treatment with saline (ECM-SAL) or Ozagrel (ECM-OZA). TxB2 levels were not different between the first four groups, but ECM mice that were treated with Ozagrel showed a marked (>80%) decrease in TxB2 levels in relation to ECM mice untreated or treated with saline (p < 0.0001). Data are presented as mean ± standard deviation.

Thromboxane A2 (TxA2) is one of the most potent vasoconstrictors acting in brain vessels, and also induces platelet aggregation, both events observed in ECM. We asked whether administration of Ozagrel, a thromboxane synthase inhibitor, to mice with ECM would lead to arteriolar relaxation and increased CBF. In fact, treatment of sick mice with Ozagrel (100 mg/kg) resulted in a significant increase in CBF (median: 16.8%; IQR: 10.3–23%) after 60 minutes, compared to mice receiving only vehicle (Fig. 3A). Brain levels of TxA2 (measured by detection of its derivative TxB2) were not significantly different in mice with ECM compared to uninfected controls or mice infected with a *P. berghei* strain (NK65) that does not cause ECM (“non-ECM”), although some ECM mice showed very high levels (Fig. 3B). In any case, brain TxA2 levels plummeted upon administration of Ozagrel to mice with ECM (an 80% decrease compared to mice with ECM before treatment or after treatment with saline) (Fig. 3B).

### Combined administration of L-arginine and Ozagrel has no additive or synergistic effects

**Figure 4:**
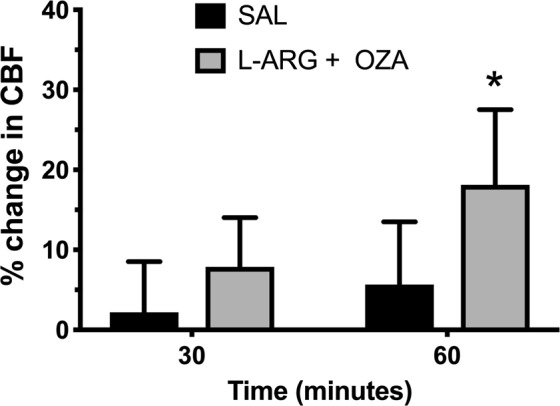
Cerebral blood flow (CBF) of mice with experimental cerebral malaria (ECM) treated with L-arginine and Ozagrel combined. Percent change in CBF in mice with ECM treated with L-arginine + Ozagrel (L-ARG + OZA, 50 mg/kg and 100 mg/kg, respectively) or vehicle (saline – SAL), 200 μL, subcutaneously. Mice were anesthetized with urethane, kept at 36 °C, had the skull exposed and were subjected to LSCI measurements of CBF before and continuously after dosing, for 60 minutes. Mice with ECM that received the combination of L-arginine and Ozagrel (n = 7) showed higher increases in CBF than mice that received saline (n = 6), at 60 minutes (p = 0.0463). Data are presented as median ± interquartile range.

Because both Ozagrel and L-arginine induced marked increases in CBF after 60 minutes in mice with ECM, we asked whether combination of the two compounds would have any additive effect. The combination in fact induced an increase in CBF, however it was not superior to each compound administered alone (Fig. 4).

### Treatments with Ozagrel or L-arginine combined with artesunate do not reverse breakdown of the blood-brain barrier (BBB) in mice with ECM

**Figure 5:**
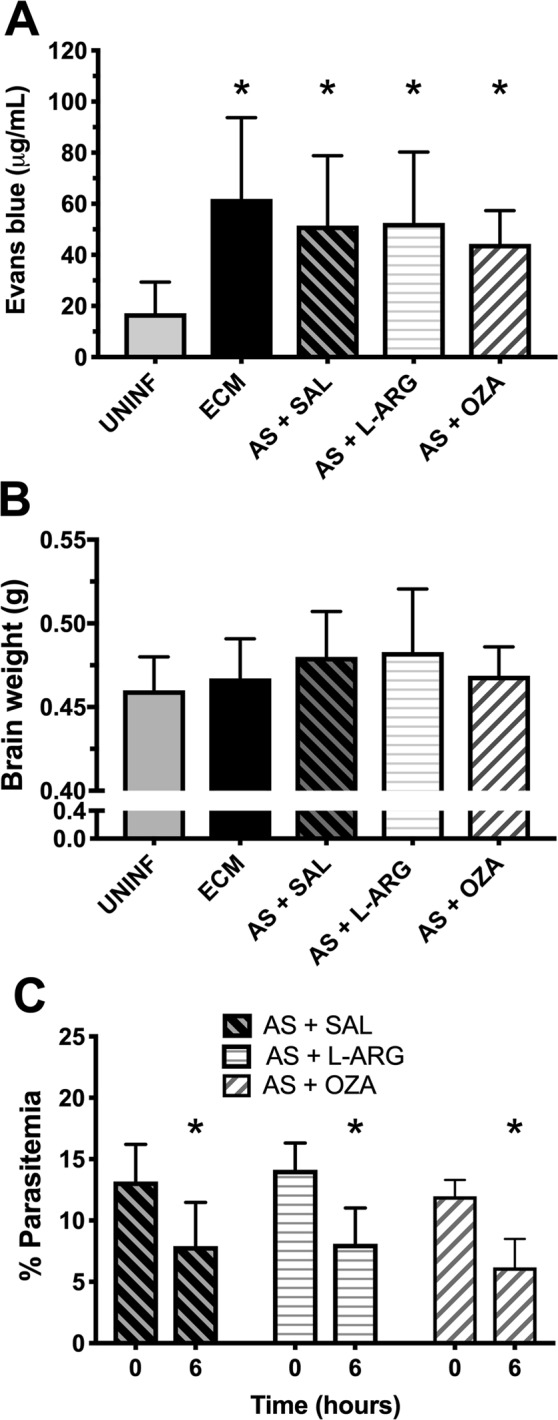
Blood brain barrier permeability, brain weight and parasitemia in mice treated with artesunate plus L-arginine or Ozagrel. (**A**) Blood brain barrier permeability measured by concentration of extravasated Evans blue dye in brain tissue. Evans blue dye was intravenously injected in mice 5 hours after treatment and allowed to circulate for one hour and then mice were subjected to euthanasia and the brain harvested. All groups, mice with ECM untreated (ECM) or treated with artesunate (AS) plus either saline (SAL), L-arginine (L-ARG) or Ozagrel (OZA), showed increased permeability (high levels of Evans blue) compared to uninfected control mice (UNINF) (p < 0.0001) (n = 8–15 mice per group). There were no differences between the infected groups, treated or not. (**B**) Wet brain weight of the brain of uninfected and mice with ECM untreated or treated with artesunate plus saline, L-arginine or Ozagrel. No significant differences in brain weight were observed between the groups. (**C**) Effect of artesunate treatment on the parasitemia of mice with ECM treated with artesunate plus saline, L-arginine or Ozagrel. In all groups, artesunate led to a reduction of around 40% in parasitemia after 6 hours. Data are presented as mean ± standard deviation.

Mice with ECM showed increased Evans blue dye extravasation to the brain 6 h after saline administration, indicating breakdown of the BBB (Fig. 5A). Treatment with artesunate plus Ozagrel or L-arginine did not have any effect on dye extravasation (Fig. 5A). No significant changes in wet brain weight were observed in mice with ECM before or after treatment with artesunate plus saline, L-arginine or Ozagrel, in relation to uninfected control mice (Fig. 5B). Artesunate was capable of decreasing parasitemia by nearly half in all treated groups 6 hours after treatment (Fig. 5C).

### Treatment with L-arginine in combination with artesunate improves CBF and prevents aggravation of cerebral ischemia in mice with ECM

**Figure 6:**
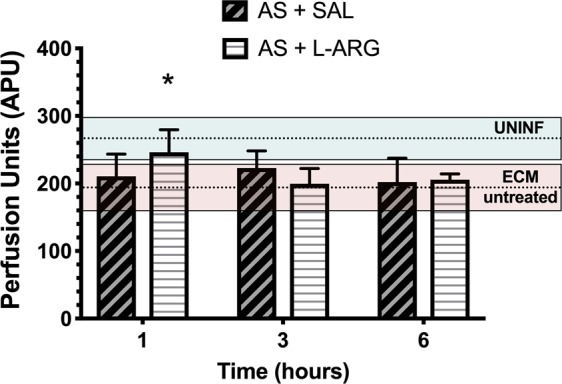
Cerebral blood flow (CBF) in mice with ECM treated with artesunate plus L-arginine or saline. CBF (measured as arbitrary perfusion units, APU) in mice with ECM treated with artesunate (AS) 32 mg/kg, intraperitoneally and either L-arginine (L-ARG) 50 mg/kg or vehicle (saline – SAL). Mice were anesthetized with urethane, kept at 36 °C, had the skull exposed and were subjected to LSCI measurements of CBF. Measurements were made in separate groups of animals 1 hour, 3 hours and 6 hours after treatment, and shown as bars in the figures (n = 4–8 mice per group). The mean ± standard deviation of CBF of uninfected control mice (UNINF) or mice with ECM untreated are shown as dotted lines (mean) and coloured horizontal boxes (standard deviation; pale green for uninfected controls and pale red for mice with ECM), to allow comparison throughout the timepoints. Comparisons were made between the treated groups (SAL versus L-ARG) at each timepoint. Mice treated with artesunate plus L-arginine showed higher CBF than mice treated with artesunate plus saline at 1 hour (p = 0.0262). Data are presented as mean ± standard deviation.

Since L-arginine showed better performance in increasing CBF in mice with ECM, we asked whether this effect could be sustained in a setting of adjuvant therapy with artesunate. Mice with ECM were treated with artesunate (32 mg/kg, intraperitoneal) and either saline or L-arginine 50 mg/kg and CBF was checked at 1 hour, 3 hours and 6 hours. At the 1-hour timepoint, animals that received artesunate plus L-arginine showed increased CBF (mean 25.2 ± 13%) in relation to ECM mice before treatment, and CBF in this group was significantly higher than in mice receiving artesunate plus saline (Fig. 6). Indeed, CBF of ECM mice treated with artesunate plus L-arginine at 1 hour was not different from that of uninfected controls, showing that the treatment reversed ischemia. The effect of L-arginine treatment was transient, as at the 3- and 6-hour timepoints CBF returned to pre-treatment levels and was not different from animals that received saline.

### Mice with ECM show increased levels of vasoconstrictor AA metabolites in the brain

**Figure 7:**
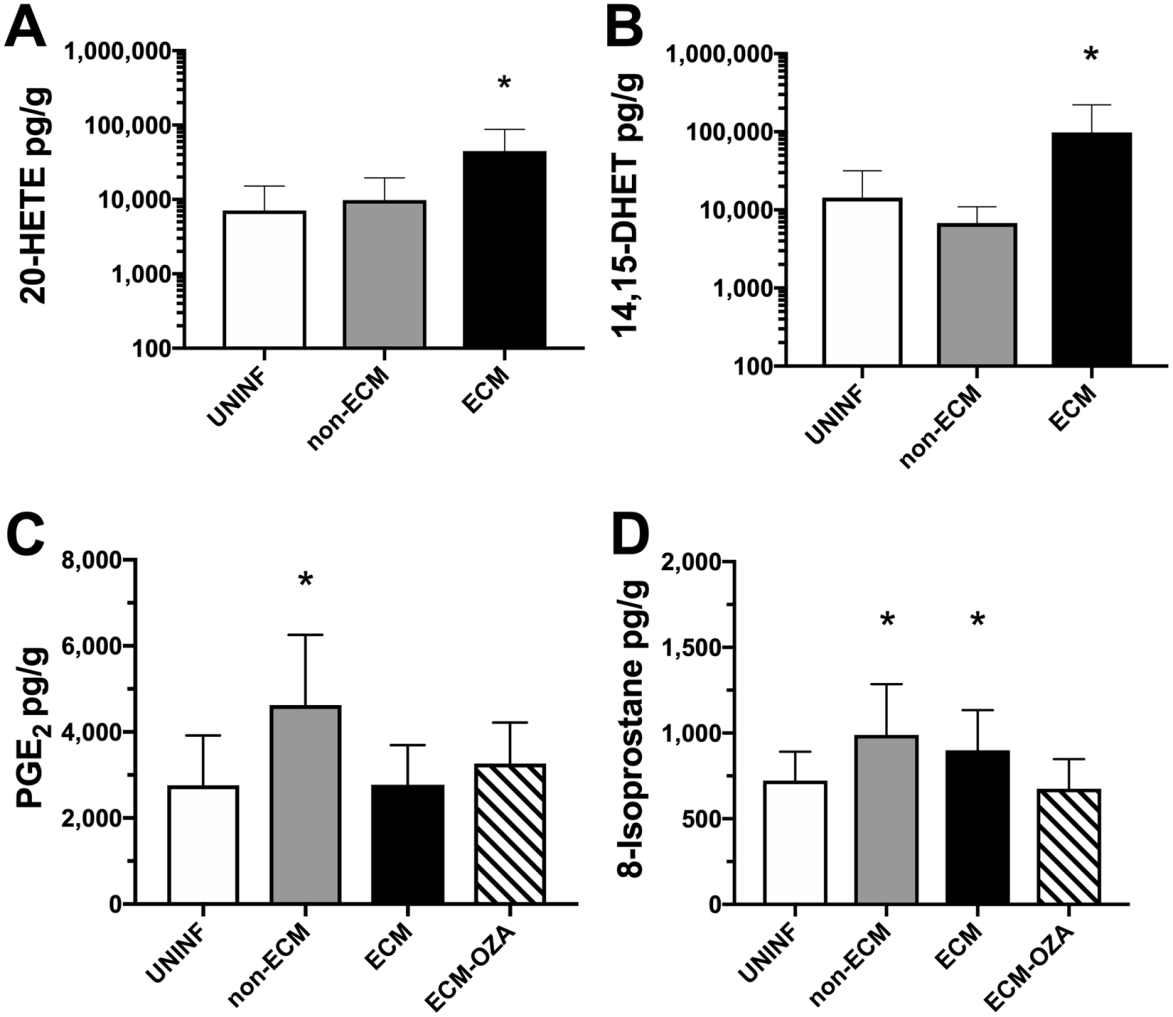
Levels of arachidonic acid metabolites in the brain of mice with ECM. Uninfected control mice (UNINF), mice infected with *P. berghei* NK65 (non-ECM) and mice with ECM were subjected to euthanasia and had the brains harvested and processed for measurement of selected arachidonic acid metabolites (20-HETE, 14,15-DHET, PGE2 and 8-isoprostanes) by ELISA. In the case of PGE2 and 8-isoprostanes, brain samples from mice with ECM treated with Ozagrel (OZA) were also assayed (n = 11–20 mice per group, in two independent experiments). (**A**) Mice with ECM showed marked increases in the brain levels of 20-HETE in relation to uninfected control and non-ECM mice (p = 0.0031). (**B**) Mice with ECM showed marked increases in the brain levels of 14,15-DHET in relation to uninfected control and non-ECM mice (p = 0.0005). (**C**) Non-ECM mice showed increased levels of PGE2 in relation to uninfected controls and mice with ECM (p = 0.0274). ECM mice treated with Ozagrel (ECM-OZA) showed PGE2 levels similar to uninfected controls. (**D**) Mice with ECM and non-ECM showed increased levels of 8-isoprostanes in relation to uninfected controls (p = 0.0055). There was no difference between the levels of 8-isoprostanes between ECM and non-ECM mice. Mice with ECM treated with Ozagrel showed 8-isoprostane levels similar to uninfected controls. Data are presented as mean ± standard deviation.

As described above, TxA2 levels were not changed in mice with ECM, but inhibition of TxA2 production by Ozagrel in these mice induced substantial increases in CBF (Fig. 3). We asked whether the cerebrovascular constriction observed in ECM could be associated with a dysregulation of other AA metabolites in the brain. Indeed, mice with ECM showed marked increases in the levels of the potent vasoconstrictor 20-HETE (Fig. 7A) and also of 14,15-DHET (Fig. 7B) in relation to uninfected controls, whereas the levels of these AA metabolites did not change significantly in mice infected with *Plasmodium berghei* NK65, which does not cause cerebral malaria (non-ECM mice). On the other hand, brain levels of the vasodilator AA metabolite PGE2 were increased in the brain of non-ECM mice and unchanged in mice with ECM in relation to uninfected controls (Fig. 7C). Brain levels of 8-isoprostanes, which are vasoconstrictor AA metabolites generated non-enzimatically by oxidative stress, were increased in mice with ECM as well as in mice infected with PbNK65 compared to uninfected animals (Fig. 7D). We also asked whether inhibition of thromboxane synthase (which, like PGE2 synthase, uses PGH as substrate) by Ozagrel might influence the levels of PGE2 and 8-isoprostanes. Ozagrel treatment did not affect the levels of PGE2, but led to decreased levels of 8-isoprostanes (Fig. 7C,D). Overall, these findings suggest that mice with ECM indeed present a shift in AA metabolism favouring the production of vasoconstrictor over vasodilator metabolites, and that a better balance is achieved by mice infected with a non-ECM-inducing parasite.

## Discussion

Cerebral malaria is a severe vasculopathy characterized by phenomena such as blockade of the cerebral microcirculation by pRBCs and leukocytes, inflammation, endothelial activation and dysfunction, ischemia/hypoxia and breakdown of the blood-brain barrier with resulting edema. We have previously shown that ECM is associated with a process of cerebrovascular constriction that limits CBF, and that this process is characterized by low NO bioavailability and endothelial and neuronal nitric oxide synthase (NOS) dysfunction^17,19,22,32^. Recently, we have shown that direct superfusion of the NOS substrate L-arginine on pial vessels of mice with ECM induced immediate dilation. Moreover, systemic administration of L-arginine, combined or not with artesunate, was shown to prevent aggravation of cerebrovascular constriction. These benefits of L-arginine on cerebral microcirculation were associated with increased survival^23^.

In the present study we showed that the previously observed pial vasoconstriction in mice with ECM was indeed paralleled by a marked decrease in CBF. In human CM, cerebral ischemia has been suggested by findings of vascular occlusion mainly in post-mortem studies and also in retinoscopy and magnetic resonance imaging (MRI) analyses^33–35^. *Plasmodium berghei* ANKA-infected mice develop a neurological syndrome (ECM) that resembles more the pediatric than the adult human CM, with vascular occlusion, brain edema and breakdown of the blood brain barrier^18,36^. Impaired brain perfusion has been shown in ECM using MRI and brain intravital microscopy^18,19,23^. Therefore, vascular dysfunction and occlusion leading to ischemia seem to be critical factors in the pathogenesis and lethality of cerebral malaria, and interventions to restore cerebral perfusion are expected to impact patient’s survival and recovery. In human severe and moderately severe malaria, infusion of L-arginine improved peripheral vascular responses and increased NO bioavailability^13–15,37^. Limitations of human CM studies, however, makes it difficult to assess the vascular and blood flow responses where it is highly relevant, in the brain. Therefore, the finding of the present study that L-arginine infusion indeed results in immediate increases of CBF in ECM is of critical importance.

When used in combination with artesunate as adjunctive therapy to ECM, again L-arginine was found to induce increased CBF in the short-term (1 hour) in mice with ECM, actually restoring cerebral perfusion to levels not different from uninfected controls. The increase in CBF subsided 3 and 6 hours after treatment. Although transient, this initial reversal of cerebral ischemia may help the very sick mice with late-stage ECM to preserve brain function better than mice receiving only artesunate. Indeed, this effect of L-arginine on CBF is associated with increased survival^23^.

NO is a central regulator of vascular tone and therefore an obvious target in diseases running with low NO bioavailability and vascular dysfunction such as cerebral malaria. However, regulation of CBF in the brain is highly complex and a number of mechanisms act in concert affecting vascular tone^24,38,39^. Astrocytes play a central role in this process. In physiological conditions, following signalling by neuron-derived glutamate, metabotropic receptors on astrocytes are engaged, increasing intracellular Ca^+ +^ and activating phospholipase A2 (PLA2), which releases arachidonic acid (AA) from membrane phospholipids. AA can then be processed by different enzymes such as ciclooxygenases, lipoxygenases, CYP450 epoxygenases, and downstream by others such as thromboxane synthase, leading to the generation of vasoactive derivatives. Depending on the pathways that are activated, vasodilator or vasoconstrictor responses will ensue leading to respectively increased or decreased blood flow to the specific area of the brain where the response is taking place^24,25^. In ECM, cerebrovascular constriction leading to ischemia is observed at the late stages of the disease. In addition to low NO bioavailability, it has been shown that potent vasoconstrictors such as endothelin-1 are increased in ECM^40^, and that vasoconstrictor leukotrienes were deleterious, whereas vasodilator prostaglandins were beneficial, in ECM^30,31^. These findings suggest that ECM pathogenesis might disturb AA metabolism in the brain, leading to predominant generation of vasoconstrictor over vasodilator AA metabolites, and that this disturbance would contribute to the decreased CBF observed in these animals. The data presented in present study are in agreement with this assumption.

We first explored the effect of thromboxane synthase inhibitors in the brain thromboxane A2 (TxA2) concentration and in CBF of mice with ECM. TxA2 is a potent vasoconstrictor produced by astrocytes as well as by activated platelets. Little is known about any role of TxA2 in ECM pathogenesis, although an early hypothesis proposed that TxA2 would exert deleterious effects in malaria through its vasoconstrictor and platelet aggregation properties whereas prostacyclin, with the opposite profile, would be protective^41^. A recent study with a limited number of plasma samples from uncomplicated and severe malaria patients indicated that TxA2 plasma levels were lower in malaria than in uninfected healthy controls^42^. However, plasma levels of TxA2 may be more representative of systemic production by platelets, whereas the local astrocyte source may be more relevant for the brain levels as measured here. Our data showed that mice with ECM present variable levels of TxA2 in the brain, in some cases very high levels, but overall no significant differences were observed when compared to uninfected controls or mice with non-cerebral malaria. However, systemic administration of the thromboxane synthase inhibitor Ozagrel caused a marked decrease in brain TxA2 levels in mice with ECM and this was associated with increased in CBF as measured by LSCI. These data suggest that blocking even the basal TxA2 generation in the brain can be of benefit to reverse vasoconstriction and restore blood flow in mice with ECM. In addition, it is possible that Ozagrel may present off-target effects and induces increased CBF by mechanisms other than thromboxane synthase inhibition.

The AA-related pathways of CBF control do not act independently, but in a balance between their own products and in concert with a number of other pathways, including NO production by endothelial cells and neurons^24^. NO influences and is influenced by different AA metabolites. For instance, NO inhibits 20-HETE synthesis and therefore induces vasodilation by this mechanism^43^. Also, low NO bioavailability can be associated with activation of thromboxane receptors, and NOS inhibition increases TxA2 production^44,45^. Conversely, increased NO production suppresses TxA2 release. We therefore asked whether combining the treatments targeting different pathways (increasing NO with L-arginine and decreasing TxA2 with Ozagrel) would result in an additive or synergistic effect. However, the combination resulted in an increase in blood flow that was not better than each treatment alone. This finding may be explained by the very fact that the different pathways of CBF regulation actually interact and are redundant and complementary.

Low NO bioavailability and increased levels of TxA2 affect vascular diameters, inducing vasoconstriction, and can also lead to increased vascular permeability and breakdown of the blood-brain barrier, a hallmark of ECM^36^. We asked whether L-arginine or Ozagrel treatments might have a benefit on this parameter, in addition to the effects on CBF. However, ECM mice treated with artesunate and either L-arginine or Ozagrel showed cerebral vascular leakage of Evans blue dye similar to untreated ECM mice or ECM mice treated with artesunate and saline indicating no benefit effect of these drugs on breakdown of the blood-brain barrier.

In addition to CBF and blood-brain-barrier permeability, L-arginine supplementation may affect many other parameters that were not assessed in this study. For instance, in addition to NO synthesis it may affect the urea cycle as well as protein and polyamine synthesis, potentially affecting ECM treatment outcome. Polyamines, for instance, have been shown to be important for malaria parasites and also in the physiology and metabolism of the nervous system^46,47^. Both human and mouse cerebral malaria cause depletion of arginine, citrulline and ornithine, due to a marked decrease in arginine appearance^9^. It is possible that ornithine depletion can also result from increased conversion to polyamines, proline and glutamate, and these pathways may be affected by L-arginine supplementation. Further research is needed to explore these mechanisms.

Since thromboxane synthase inhibition was shown to increase CBF in mice with ECM, we assessed how the disease affected the levels of other relevant vasoactive AA metabolites. First, mice with either ECM or non-cerebral malaria presented increased levels of 8-isoprostanes compared to uninfected controls. The 8-isoprostanes are generated by non-enzymatic oxidation of AA^48,49^, therefore malaria infection per se induced significant oxidative stress. Increased plasma and urine levels of 8-isoprostanes have been reported in human and mouse severe malaria^50–52^. 8-isoprostanes are recognized as potent vasoconstrictors^53–55^ and, interestingly, cause toxicity on endothelial cells, which was related to increased TxA2 formation and was prevented by thromboxane synthase inhibitors^56^. This is consistent with the finding in this study that treatment of mice with ECM with the thromboxane synthase inhibitor Ozagrel led to decreased levels not only of TxA2 itself but also of 8-isoprostanes. The mechanism by which Ozagrel causes a decrease in 8-isoprostane levels in ECM, however, is not clear. Since 8-isoprostanes are generated non-enzymatically by oxidative stress^48,49^, improved CBF and hence improved tissue oxygenation and washout of metabolic waste resulting from Ozagrel treatment may contribute to decreasing oxidative stress and 8-isoprostane formation in the brain. Further research is needed to address these possibilities, and also to determine whether Ozagrel and also L-arginine treatments affect the levels of other AA metabolites such as 20-HETE and 14,15-DHET.

Since both ECM and non-ECM mice showed increased levels of brain 8-isoprostanes, it appears that this mediator cannot per se be associated with the pathogenesis of ECM, or in the cerebral vasoconstriction observed in this syndrome^19^. Nevertheless, the brain levels of the enzymatically-generated AA derivatives indicate that in ECM the balance leans towards a vasoconstrictor over a vasodilator response, and the opposite occurs in non-ECM. ECM mice showed increased levels of 20-HETE and 14,15-DHET, while keeping levels of PGE2 unchanged. Conversely, non-ECM mice showed increased levels of PGE2 and no significant changes in 20-HETE and 14,15-DHET levels.

The profile of PGE2 response - increased in non-ECM and unchanged in ECM - is in line with previous studies showing that prostaglandins are protective in ECM. One study showed that mice with ECM had increased phospholipase A2 mRNA expression in the spleen, and that treatment of mice with the prostaglandin synthesis inhibitor aspirin was detrimental^30^. Another study showed that increased COX-1 expression was observed in models of cerebral and non-cerebral malaria, whereas increased COX-2 expression in the brain was observed only in mice with ECM^31^. As in the case of treatment with aspirin, treatment with the COX-2 inhibitor celecoxib was detrimental. In human malaria, lower levels of PGE2 were also correlated with disease severity^28^, and reduced PGE2 was shown to occur through hemozoin-induced inhibition of COX-2 in mononuclear cells via an interleukin-10-dependent mechanism^29^. PGE2 is an important inflammatory mediator, but it is also a key vasodilator molecule produced to regulate blood flow in the brain. Therefore, the increased levels of PGE2 observed in non-ECM but not in ECM mice could be protective, helping to prevent cerebral vasoconstriction.

The brain levels of 14,15-DHET were increased in mice with ECM, but not in non-ECM. The magnitude of the increase was substantial, with ECM mice showing on average several fold increases in relation to non-ECM mice and to uninfected controls. 14,15-DHET is a major metabolite of epoxy-eicosatrienoic acids (EETs), which are produced by the conversion of AA by CYP450 in astrocytes. EETs are vasodilator AA metabolites, with critical roles in the regulation of CBF in physiological conditions^57^. EETs are metabolized by soluble epoxide hydrolases (sEH) to inert products such as 14,15-DHET. Increased activity of sEH, generating increased levels of DHETs, therefore prevents the vasodilator action of EETs, resulting in vasoconstriction^55^. Indeed, patients with vascular cognitive impairment were shown to exhibit increased sEH expression and increased levels of 14,15-DHET in the cerebral microcirculation^58^.

Finally, the levels of the potent vasoconstrictor 20-HETE were also markedly elevated in mice with ECM. Whether low NO bioavailability, known to occur in ECM, plays a role in generating high levels of 20-HETE, as observed in this study, as it prevents physiological inhibition of CYP4A, or it is the opposite, that is, ECM induces increased production of 20-HETE that in turn promotes low NO bioavailability and endothelial dysfunction, or both, remains to be demonstrated. But all these data are consistent with a pro-vasoconstrictor ambiance in ECM.

In summary, the data presented here further confirm that L-arginine supplementation results in transient but relevant reversal of cerebral ischemia in ECM. The data also demonstrate that the balance of selected vasoactive AA metabolites, produced in the neurovascular unit and known to play key roles in the regulation of CBF, is altered in mice with ECM, assuming a vasoconstrictor profile, whereas non-ECM mice present a vasodilator profile. Targeted inhibition of a selected AA-derived vasoconstrictor, thromboxane A2, led to immediate increase in CBF, which was not further incremented by concomitant NO-inducing treatment strategy (L-arginine). The data presented here indicate that more in-depth characterization of neurovascular unit alterations in ECM and its manipulation may provide new insights on ECM pathogenesis and therapeutics, particularly in relation to vascular pathology.

## Material and Methods

### Animals

Eight-to-ten-week-old female C57BL/6 mice (18–20 g) from the Fundação Oswaldo Cruz (FIOCRUZ) breeding unit (ICTB) were used for the studies. The animals were kept at constant temperature (22–24 °C) with free access to chow and water in 12-hour light/dark cycle. All experiments were performed in accordance with relevant guidelines and regulations. The Animal Welfare Committee of the FIOCRUZ under license number LW33/10, LW29/13 and L-037/15 (CEUA/FIOCRUZ) approved the experiments in these studies.

### Parasites and infection

C57BL/6 red blood cells infected with *Plasmodium berghei* ANKA expressing the green fluorescent protein (PbA-GFP, a donation from the Malaria Research and Reference Reagent Resource Center-MR4, Manassas, VA; deposited by C.J. Janse and A.P. Waters; MR4 number: MRA-865) or *Plasmodium berghei* NK65 (PbNK65) previously kept in liquid nitrogen were thawed and intraperitoneally (i.p.) inoculated into healthy mice that served as parasite donor for the infection of experimental groups. Eight-to-ten-week-old female C57BL/6 mice were intraperitoneally inoculated with 1 × 10^6^ red blood cells parasitized by PbA (PbA-GFP pRBC) or with 1 × 10^6^ PbNK65 pRBC (day 0 of infection). Parasitemia was checked by microscopical examination of Giemsa-stained blood smears or by flow cytometry (PbA-GFP). For rectal temperature measurement, a thermocouple probe (Oakton® Acorn TM; Oakton Instruments, IL, USA) was used. On day 6 post-infection, PbA-infected animals with rectal temperature range between 31 and 36 °C were used in the study^19^.

### Treatments

A first set of experiments was designed to verify the short-term (1 h), immediate CBF responses to each drug (L-arginine, Ozagrel, isolated or combined) or control vehicle (saline) in mice with late-stage ECM. On day 6 after inoculation, parasitemia and rectal temperature were checked. Mice presenting hypothermia (31–36 °C), which indicates late-stage ECM, were randomly assigned to drug (L-arginine and/or Ozagrel) or control (saline) groups. The parasitological (parasitemia) and clinical (rectal temperature) data were compared a posteriori between groups to verify eventual bias in group assignment. Mice received L-arginine (Tocris Bioscience, Ellisvile, MO, USA) 50 mg/kg, and/or the thromboxane synthase inhibitor Ozagrel (Tocris Bioscience) 100 mg/kg, in saline, in final volume of 200 μL, subcutaneously (s.c.). Control mice received 200 μL of saline. For the assessments (CBF and vascular permeability) requiring longer delays (1–6 hours) between treatment and measurements, L-arginine, Ozagrel or saline were given as described above, in combination with artesunate (Sigma) given intraperitoneally at 32 mg/kg in 5% sodium bicarbonate solution. The dose and treatment scheme for artesunate were defined in a previous study^59^. Since continuous intravenous infusion, as done in humans, is not practical in mice, a higher single daily intraperitoneal dose of artesunate was implemented to achieve fast parasite clearance.

### Analysis of microvascular cerebral blood flow

Microvascular cerebral blood flow was evaluated using a laser speckle contrast imaging system (LSCI) (Perimed, Jarfalla, Sweden). LSCI provides a perfusion index proportional to the concentration and mean velocity of red blood cells. This methodology allows to quantify cortical blood flow changes with excellent spatial and temporal resolution^60,61^. On day 6 post-infection animals with rectal temperature range between 31 and 36 °C were anesthetized with urethane (Sigma, USA) 2 mg/g i.p.. Mice had their scalp retracted and the skull exposed and were positioned under a LSCI light with a 785 nm wavelength. The body temperature of animals was kept at 36 °C using a thermostatic heating pad (Harvard Apparatus). The distance between the laser light source and the skull was 10 cm as recommended by the manufacturer. To assess the microvascular cerebral blood flow in real time, a region of interest (ROI) was defined, covering almost the entire area available for imaging, and the same ROI was used for all the animals used in the study. For the short-term (1 h) assessments, measurements of cerebral blood flow were performed before (baseline) and after the intervention (treatment) continuously for 60 minutes. In case of longer term (3 h and 6 h) assessments, different groups of mice were used for each timepoint. Analysis of six laser speckle images per second and relative cerebral blood flow of all animals were acquired using Perisoft software (Perimed, Jarfalla, Sweden) and expressed as arbitrary perfusion units (APU).

### Brain eicosanoids extraction and determination of their concentrations by Enzyme-linked immunosorbent assays (ELISA)

PbA-infected animals with ECM and PbNK65-infected mice were subjected to euthanasia and had their brains harvested on day 6 post-infection. Similarly, groups of mice with ECM treated with Ozagrel 100 mg/kg or saline had their brains harvested one hour after the treatment. Brains from uninfected mice were used as control. The samples were immediately flash frozen in liquid nitrogen and stored at −70 °C until processed. For eicosanoids determination, the brain was thawed, weighed and then homogenized on ice with 1 mL of homogenization buffer [0.1 M sodium phosphate, pH 7.4, containing 1 mM of ethylenediamine tetraacetic acid (EDTA) and 10μM indomethacin] using a tissue grinder (Corning). Samples were centrifuged at 20,000 *g* for 30 min at 4 °C. The resulting supernatant (approximately 700 µL) was collected and mixed with the same volume of ethyl acetate (Vetec) and acetonitrile (Vetec) mixture (1:1) and then vortexed for 45 seconds. Samples were centrifuged again at 10,000 *g* for 15 min at 4 °C. The resulting organic phase was collected, dried using a speed vaccum (model iss110, Savant) for 3 hours and frozen at −70 °C. On the day of the assay, the dry samples were resuspended in 500 μL of enzyme immunoassay (EIA) buffer and the levels of TXB_2_, 8-isoprostanes and PGE_2_ were measured using EIA kits from Cayman Chemical and the levels of 20-HETE and 14,15-DHET were measured using ELISA kits from Detroit R&D, according to the manufacturers’ instructions.

### Blood-brain barrier permeability (Evans blue dye) assay

Animals with ECM were treated with artesunate (32 mg/kg i.p.) and either saline s.c., L-arginine (50 mg/kg s.c.) or Ozagrel (100 mg/kg s.c.). Five hours later animals were anesthetized with urethane (2 mg/g i.p.) and injected intravenously through orbital sinus with 150 µL of a 2% solution of Evans blue dye (Sigma) in PBS 1×. The dye was allowed to circulate for 1 hour and then the mice were transcardiacally perfused with ice-cold saline (10 mL) through the left ventricle. Animals were euthanized by decapitation, the brain was harvested, weighed and then incubated in 3 mL of formamide (Sigma) for 48 hours at 37 °C. The amount of Evans blue dye in the formamide solution was measured in a spectrophotometer at a wavelength of 620 nm.

### Statistical analyses

All statistical analyses were performed using a statistical software package (Prism 7.0, GraphPad). To analyze parasitemia, body temperature and basal cerebral blood flow measurements a two-tailed unpaired t-test was used. Student t-test with Mann-Whitney correction and two-way ANOVA were used for analysis of cerebral blood flow before and after interventions. One-way ANOVA and Student t-test with Mann-Whitney correction were used for ELISA and Evans blue dye assay data analysis. Data are presented as mean ± standard deviation, except in the case of percentual changes in blood flow, where median and interquartile ranges (IQR) were used. P < 0.05 was considered statistically significant.
